# Impact of Cumulative Fluid Balance During Continuous Renal Replacement Therapy on Mortality in Patients With Septic Acute Kidney Injury: A Retrospective Cohort Study

**DOI:** 10.3389/fmed.2021.762112

**Published:** 2021-11-15

**Authors:** Jin Lin, Hai Zhou Zhuang, De Yuan Zhi, Zhili Qi, Jing Bai, Lei Dong, Shuai Liu, Meili Duan

**Affiliations:** ^1^Department of Critical Care Medicine, Beijing Friendship Hospital, Capital Medical University, Beijing, China; ^2^Department of Critical Care Medicine, Beijing Tiantan Hospital, Capital Medical University, Beijing, China

**Keywords:** sepsis, acute kidney injury, continuous renal replacement therapy, fluid balance, mortality

## Abstract

**Background:** The clinicians often use continuous renal replacement therapy (CRRT) for the fluid management of patients with septic acute kidney injury (AKI). However, there is limited knowledge of the effects of changes in fluid balance (FB) on CRRT and its association with outcomes in patients with septic AKI.

**Objective:** This study aimed to determine the association of cumulative FB (CFB) during treatment with 28-day all-cause mortality in the patients with septic AKI who require CRRT.

**Methods:** This retrospective observational study examined patients who received CRRT due to septic AKI in a mixed intensive care unit (ICU) of a tertiary teaching hospital between January 2015 and December 2018. The patients were divided into three groups—negative FB, even FB, and positive FB—based on the CFB during CRRT. The primary outcome was 28-day all-cause mortality.

**Results:** We examined 227 eligible patients and the mean age was 62.4 ± 18.3 years. The even FB group had a significantly lower 28-day mortality (43.0%, *p* = 0.007) than the positive FB group (72.7%) and the negative FB group (54.8%). The unadjusted and adjusted Cox regression models indicated that the positive FB group had an increased risk for 28-day all-cause mortality relative to the even FB group. A restricted cubic splines model indicated a J-shaped association between the CFB and 28-day all-cause mortality in the unadjusted model.

**Conclusion:** Among the critically ill patients with septic AKI who require CRRT, those with positive FB had a higher mortality rate than those with even FB.

## Introduction

Sepsis is the leading cause of acute kidney injury (AKI) in intensive care units (ICUs). A septic AKI is increasingly recognized as a common and serious problem in critically ill patients, particularly in the ICU, and septic AKI occurs in about 50% of the critically ill patients with sepsis (1, 2). The previous studies reported that the mortality of ICU patients with septic AKI was 30–45% (1, 3–5), and the mortality rate for those who required renal replacement therapy (RRT) was 56–70% (6–8).

The mortality of patients with septic AKI is associated with several factors, such as AKI severity, multiple organ failure, and fluid accumulation (9, 10). Effective fluid resuscitation is crucial for the stabilization of sepsis-induced tissue hypoperfusion or septic shock (11). However, over time the initial benefit of fluid therapy can lead to fluid accumulation and tissue edema, and this can exacerbate organ dysfunction (12). Several observational studies found an association between the positive fluid balance (FB) and poor outcomes in critically ill patients with septic AKI (9, 13–15). Optimizing fluid status is essential for patients with excess fluid accumulation but is difficult to achieve when the patients develop AKI.

The clinicians often use continuous renal replacement therapy (CRRT) for fluid management of the patients with severe AKI (16, 17), although determining the appropriate fluid volume in those patients during CRRT is challenging. Some studies demonstrated that a positive FB after CRRT initiation was associated with an unfavorable outcome (18–20), but others reported that active fluid withdrawal using RRT in critically ill patients was associated with poorer survival than standard care (21). Compared with the patients with non-septic AKI, the patients with septic AKI and septic shock may have different responses to the RRT due to differences in pathophysiology (22). However, there is limited knowledge about the effects of changes in the FB on CRRT and its association with outcomes in patients with septic AKI.

This study aimed to examine the association between cumulative FB (CFB) during treatment with CRRT and 28-day all-cause mortality in critically ill patients with septic AKI. Considering that an even FB is more similar to the normal physiological state, we used a group of patients with even FB as a comparator. We hypothesized that a positive or negative FB is associated with the increased 28-day all-cause mortality.

## Methods

### Study Population

This retrospective study was conducted in a 30-bed medical-surgical ICU of a tertiary teaching hospital in Beijing, China (Beijing Friendship Hospital, Capital Medical University). A retrospective review of the medical records of patients admitted to this ICU from January 2015 to December 2018 was performed. The patients included were those admitted to the ICU with septic AKI and undergoing CRRT. Septic AKI was defined as the simultaneous presence of sepsis and AKI. The patients with the following characteristics were excluded: pre-existing chronic kidney disease (estimated glomerular filtration rate [GFR] < 20 ml/min/1.73 m^2^ for at least 1 year); ICU stay of less than 48 h; and missing data on the fluid status and body weight. Data were collected only from the first ICU admission if a patient underwent multiple ICU admissions that required CRRT during the study period. This study was approved by the Bioethics Committee of Beijing Friendship Hospital, Capital Medical University (2020-P2-210-01).

### Definitions

The definition of AKI was based on the Kidney Disease: Improving Global Outcomes (KDIGO) clinical practice guidelines for AKI. Thus, AKI was defined by the presence of at least one of the following three criteria: an increase in the serum creatinine (sCr) level to at least 0.3 mg/dl (26.5 μmol/L) within 48 h; an increase in the sCR level to at least 1.5 times the baseline level that was known or was presumed to have occurred within the previous 7 days; or urine volume below 0.5 ml/kg/h for 6 h (23). The diagnostic criteria for sepsis and septic shock were in accordance with the 2016 International Sepsis Definitions (24). The baseline eGFR was calculated by the Chronic Kidney Disease Epidemiology Collaboration (CKD-EPI) formula (25). This eGFR calculation used the sCr value closest to the date of hospitalization, but not more than 1 year prior to the hospitalization, or the lowest sCr value documented during the current hospitalization if no other value was available. Chronic kidney disease (CKD) was defined as the eGFR below 60 ml/min/1.73 m^2^.

### Determination of CFB

For each patient, the CFB was expressed as a percentage (%) and calculated using the following equation.


$$\begin{array}{l} \text{Weight}-\text{adjusted CFB }\left(\text{\%}\right)\\ =\frac{\left(Cumulative\;daily\;fluid\;input-output\right)\;in\;liters\;\times 100}{ICU\;admission\;weight\left(kg\right)\;} \end{array}$$

The CFB was assessed at 48 and 72 h after initiation of CRRT. A negative FB was defined as a weight-adjusted CFB less than 0%, an even FB as a weight-adjusted CFB of 0% to less than 5%, and a positive FB as a weight-adjusted CFB of 5% or more (26). In addition, we collected data on input and output from the hospital admission to CRRT initiation to calculate the CFB and weight-adjusted CFB at the initiation of CRRT.

### Clinical Outcomes

The primary outcome was all-cause mortality at 28 days after CRRT initiation. The secondary outcomes were all-cause mortality at 60 days, length of stay in the ICU, mechanical ventilation-free days, and vasopressor-free days within 28 days from the CRRT initiation.

### Data Collection

At baseline, the following characteristics of the enrolled patients were recorded at the time of CRRT initiation: demographic data (age, sex, body weight, and body mass index [BMI]); clinical data (admission type, comorbidities, septic shock, the CFB before CRRT initiation, and indication for CRRT); infection data (site of infection and infection category); laboratory data (white blood cells, hemoglobin, platelets, eGFR, sCr, albumin, and lactate). For assessment of disease severity, the Sequential Organ Failure Assessment (SOFA) score and the Acute Physiology and Chronic Health Evaluation (APACHE) II score were determined at the time of CRRT initiation. The organ support measures at the time of CRRT initiation, such as the need for mechanical ventilation and vasopressor support, were recorded.

### Statistical Analysis

For comparisons, the patients were stratified into three groups based on 48 h weight-adjusted CFB: positive FB, negative FB, and even FB. The baseline values of the continuous variables were reported as means and SDs or medians and interquartile ranges (IQR) for normally and non-normally distributed variables, respectively. The categorical variables were presented as numbers and percentages. The categorical variables were compared using the chi-squared test and continuous variables using a one-way ANOVA and the Kruskal–Wallis test.

Survival analysis was performed using a Kaplan–Meier method and the log-rank test. The multivariable Cox regression models were used to examine the association of 28-day mortality with CFB. The Cox regression was first adjusted for demographic data (age, sex, and BMI; Model 1) and then additionally adjusted for severity of illness (SOFA score and APACHE II score; Model 2). The clinical variables with *p* values below 0.03 in the univariate analysis and previous variables were entered into the final model (Model 3). If two variables were strongly correlated, only one of these variables was retained and added to the multivariable model. The weight-adjusted CFB was also treated as a continuous variable in a Cox regression model that was used to calculate the unadjusted and adjusted hazard ratio (*HR*s); these HRs were plotted using restricted cubic spline models to assess the potential nonlinear associations between the CFB and 28-day mortality.

A subgroup analysis was performed to examine primary outcomes in the patients with fluid overload before CRRT initiation and patients with septic shock. To explore the robustness of the results, three sensitivity analyses were performed. First, the multivariable logistic regression models were constructed to calculate the unadjusted and adjusted odds ratios (*OR*s) to test the robustness of the findings from the Cox models. Second, to account for potential survivorship bias, the effect of CFB on 28-day mortality in a subgroup of patients who survived at least 3 days after CRRT initiation was examined. Third, to reduce the effect of selection bias and potential confounding, a propensity score representing the probability that a patient would be in a fluid balance group was developed based on the following variables: age, sex, BMI, SOFA score, APACHE II score, septic shock, CKD, hospital-acquired infection, lactate, eGFR, and weight-adjusted CFB before CRRT. This score was calculated using logistic regression and additionally adjusted in the Cox regression model. Another propensity score was calculated using the baseline variables that differed significantly among the three groups and adjusted in the Cox regression.

Data were analyzed using IBM SPSS software version 19.0 (IBM, NY, USA), R version 4.0.1 (Austria), and STATA version 14.1 (Stata Corp LLC, TX, USA). A two-sided *p*-value below 0.05 was considered significant.

## Results

### The Baseline Characteristics

**Figure 1:**
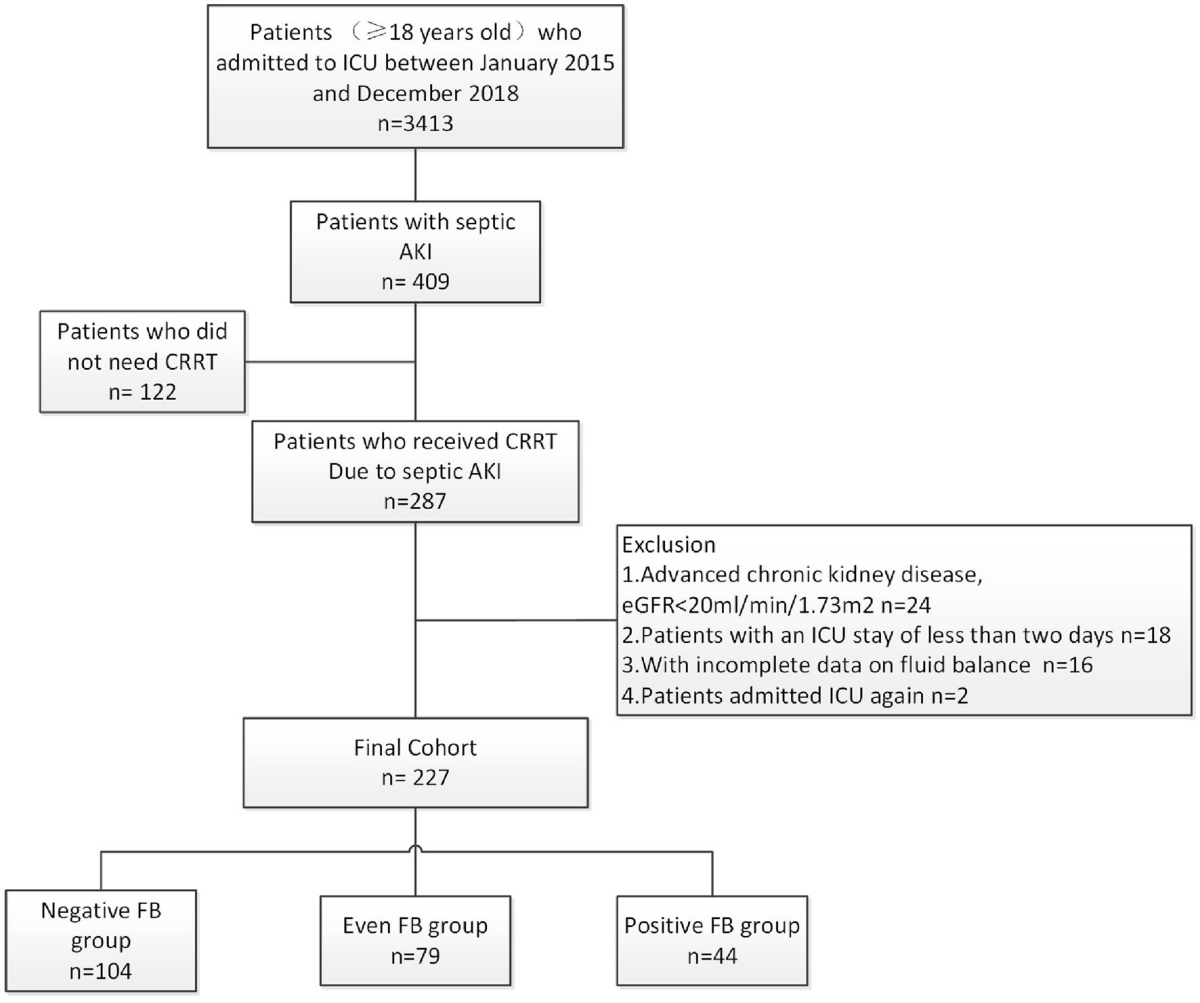
Disposition of patients who were admitted to the ICU, had septic AKI, required CRRT, and were enrolled in the different CFB groups.

During the 4-year study period, 3,413 critically ill adult patients were admitted to the ICU, and 287 patients developed severe septic AKI that required CRRT (Figure 1). After the exclusion of patients based on pre-defined criteria, we included 227 patients in this study.

We recorded the demographic, clinical, and laboratory characteristics of all the patients with stratification by CFB at baseline (Table 1). Overall, most of the patients were male (64.3%) and the mean age was 62.4 ± 18.3 years. There were 104 patients (45.8%) in the negative FB group, 79 (34.8%) in the even FB group, and 44 (19.3%) in the positive FB group. The history of CKD was more prevalent in the negative and even FB groups. The positive FB group had a higher APACHE-II score, a higher prevalence of receiving vasopressor support and septic shock, a lower level of albumin, and a higher level of lactate. The CFB at the initiation of CRRT was significantly higher in the positive FB group. The negative FB group had a lower level of hemoglobin and smaller proportions of men and patients with hospital-required infections. The most common indications for CRRT were oliguria and anuria, followed by worsening azotemia and fluid overload. There were no significant differences among the three groups.

**Table 1:** Characteristics of patients at baseline (CRRT initiation) who had different CFB status.

**Characteristic**	**All *n =* 227**	**Negative FB (*n =* 104)**	**Even FB (*n =* 79)**	**Positive FB (*n =* 44)**	***p*** **value**
Age (years)	62.4 ± 18.3	61.7 ± 19.7	62.2 ± 18.2	64.1 ± 15.1	0.76
Male sex, *n* (%)	146 (64.3)	52 (50.0)	61 (77.2)	33 (75.0)	0.001
Weight (kg)	67.4 ±14.6	65.7 ±14.0	69.7 ± 17.1	67.4 ± 9.9	0.19
BMI (kg/m^2^)	24.2 ± 5.1	23.7 ± 4.2	24.1 ± 5.1	23.5 ± 3.1	0.76
Pre-admission renal function					
^*^Baseline Cr	86.0 [66.5, 153.0]	89.5 [67.5, 162.0]	86.9 [65.7, 190.0]	80.0 [63.0, 105.0]	0.26
^*^Baseline eGFR, mL/min/1.73 m^2^	92.6 [46.0, 123.5]	82.6 [34.9, 123.9]	92.1 [39.1, 123.1]	101.4 [74.7, 122.9]	0.14
Comorbid condition, *n* (%)					
Hypertension	111 (48.9)	57 (54.8)	32 (40.5)	22 (50.0)	0.16
Diabetes	56 (24.7)	26 (25.0)	24 (30.4)	6 (13.6)	0.12
Cardiac disease	73 (32.2)	38 (36.5)	21 (26.6)	14 (31.8)	0.36
Chronic liver disease	27 (11.9)	13 (12.5)	12 (15.2)	2 (4.5)	0.21
Chronic kidney disease	55 (24.2)	29 (27.9)	21 (26.1)	5 (4.5)	0.009
Surgery admission, *n* (%)	80 (35.2)	33 (31.7)	28 (35.4)	19 (34.2)	0.41
Septic shock, *n* (%)	172 (75.8)	70 (67.3)	59 (74.7)	43 (97.7)	<0.001
Hospital-acquired infection, *n* (%)	100 (44.1)	31 (29.8)	47 (59.5)	22 (50.0)	<0.001
Site of infection, *n* (%)					<0.001
Respiratory	136 (59.9)	77 (74.0)	47 (59.5)	12 (27.3)	
Intra-abdominal	66 (29.1)	19 (18.3)	25 (31.6)	22 (50.0)	
Urinary	6 (2.6)	2 (1.9)	0 (0.0)	4 (9.1)	
Blood	9 (4.0)	2 (1.9)	3 (3.8)	4 (9.1)	
Other	10 (4.4)	4 (3.8)	4 (5.1)	2 (4.5)	
Indications for CRRT, *n* (%)					
worsening azotemia	62 (27.3)	26 (25.0)	25 (31.6)	11 (25.0)	0.56
Oligouria or anuria	148 (65.2)	53 (67.1)	61 (58.7)	34 (77.3)	0.08
Fluid overload	59 (26.0)	31 (29.8)	15 (19.0)	13 (29.5)	0.21
Electrolyte imbalance	39 (17.2)	16 (15.4)	19 (24.1)	4 (9.1)	0.08
Acid base imbalance	51 (22.5)	21 (20.2)	19 (24.1)	11 (25)	0.75
Before CRRT initiation					
Invasive mechanical ventilation, *n* (%)	149 (65.6)	67 (64.4)	49 (62.0)	33 (75.0)	0.33
Vasopressor support, *n* (%)	138 (60.8)	61 (58.7)	43 (54.4)	34 (77.3)	0.038
APACHE II score	23.6 ± 7.0	21.8 ± 5.9	24.7 ± 7.9	26.5 ± 7.0	<0.001
SOFA score	10.5 ± 3.8	10.3 ± 3.7	10.5 ± 4.2	11.3 ± 3.3	0.34
Laboratory before CRRT					
White blood cells (× 10^9^/L)	12.6 ± 7.6	12.4 ± 6.2	12.8 ± 6.6	12.7 ± 11.6	0.94
Platelets (× 10^9^/L)	94 [48,171]	126.2 ± 98.8	110.6 ± 84.4	77 [45,179]	0.72
Hemoglobin (g/L)	93.7 ± 26.3	87.2 ± 22.7	95.3 ± 26.1	106.1 ± 30.1	<0.001
Albumin (g/L)	25.7 ± 4.7	26.8 ± 3.8	26.0 ± 5.2	22.1 ± 4.2	<0.001
^**^eGFR, mL/min/1.73m^2^	20.4 [12.7, 35.2]	18.8 [10.1, 33.9]	21.2 [12.9, 42.1]	22.7 [15.9, 32.2]	0.31
Creatinine (μmmol/L)	246.6 [155.6, 394.0]	243.8 [170.1, 420.8]	264.0 [146.5, 409.4]	241.9 [179.3, 348.7]	0.71
Lactate (mmol/L)	2.2 [1.4, 5.2]	2.0 [1.1, 4.5]	1.9 [1.5, 4.2]	3.5 [1.9, 10.2]	<0.001
CFB before CRRT (ml)	3,980 [1,910, 8,379]	3,823 [1,360, 7,001]	3,688 [1,845, 7,763]	8,294 [3,864, 11,930]	0.001
Weight-adjusted CFB (%) before CRRT	5.9 [2.7, 12.8]	5.2 [2.4, 10.2]	5.7 [2.8, 12.3]	12.6 [5.3, 16.3]	0.004
Duration of treatment					
Cumulative fluid balance, mL					
48h	204 [−1,400–2,203]	−1,550 [−2,802, −680]	1,197 [488, 1,491]	5,562 [3,798, 7,089]	<0.001
72h	367 [−2,029–2,559]	−2,043 [−3,480, −1,154]	1,679 [588, 2,332]	6,607 [5,178, 8,613]	<0.001
Weight–adjusted CFB (%)					
48h	0.2 [−2.0–2.9]	−2.2 [−4.2, −1.0]	1.7 [0.8, 2.5]	7.3 [6.1, 10.6]	<0.001
72h	0.6 [−2.8–4.3]	−3.0 [−5.2, −1.7]	2.5 [0.7, 3.5]	9.3 [7.3, 10.9]	<0.001

* *Based on SCr before hospitalization*.

** *Based on SCr at CRRT initiation. FB, fluid balance; CFB, cumulative fluid balance; BMI, Body Mass Index; eGFR, estimated glomerular filtration rate; SOFA, Sequential Organ Failure Assessment; APACHE, Acute Physiology and Chronic Health Evaluation; CRRT, continuous renal replacement therapy. Here and below, continuous variables are expressed as mean ± SD or median [Q1, Q3] and nominal variables as n (%)*.

We assessed the CFB at 48 and 72 h after initiation of CRRT. At these two times, the positive FB group had median FBs of 5,562 and 6,607 ml, and the negative FB group had median FBs of −1,550 and –2,043 ml.

### FB in Survivors and Non-survivors

Among all the 227 patients, 123 patients (54.2%) died within 28 days after CRRT initiation (Table 2). The CFB at 48 h was 204 ml (range: −1,505, 1,491) in the survivors and 379 ml (range: −1,394, 3,274) in the non-survivors. The non-survivors also had a higher CFB at 72 h after CRRT initiation, but this difference was not statistically significant.

**Table 2:** Characteristics of patients at baseline (CRRT initiation) who were survivors and non-survivors at day-28.

**Characteristic**	**All (*n =* 227)**	**Survivors (*n =* 104)**	**Non-survivors (*n =* 123)**	***p*** **value**
Age (years)	62.4 ± 18.3	60.4 ± 16.9	64.0 ± 19.2	0.14
Male sex, *n* (%)	146 (64.3)	78 (75.0)	68 (55.3)	0.002
Weight (kg)	67.4 ± 14.6	70.5 ± 16.4	64.8 ± 12.2	0.003
BMI (kg/m^2^)	24.2 ± 5.1	24.5 ± 5.2	23.2 ± 3.4	0.022
Pre-admission renal function				
^*^Baseline Cr	86.0 [66.5, 153.0]	91.0 [72.7, 246.3]	83.2 [63.0, 109.4]	0.002
^*^Baseline eGFR, mL/min/1.73 m^2^	92.6 [46.0, 123.5]	80.2 [29.9, 119.8]	98.1 [73.1, 131.4]	0.002
Comorbid condition, *n* (%)				
Hypertension	111 (48.9)	56 (53.8)	55 (44.7)	0.19
Diabetes	56 (24.7)	25 (24.0)	31 (25.2)	0.88
Cardiac disease	73 (32.2)	35 (33.7)	38 (30.9)	0.67
Chronic liver disease	27 (11.9)	9 (8.7)	18 (14.6)	0.22
Chronic kidney disease	55 (24.2)	33 (31.7)	22 (17.9)	0.019
Surgery admission, *n* (%)	80 (35.2)	38 (36.5)	42 (34.1)	0.78
Septic shock, *n* (%)	172 (75.8)	65 (62.5)	107 (87.0)	<0.001
Hospital-acquired infection, *n* (%)	100 (44.1)	36 (34.6)	64 (52)	0.011
Site of infection, *n* (%)				0.31
Respiratory	136 (59.9)	56 (53.8)	80 (65.0)	
Intra-abdominal	66 (29.1)	37 (35.6)	29 (23.6)	
Urinary	6 (2.6)	2 (1.9)	4 (3.3)	
Blood	9 (4.0)	5 (4.8)	4 (3.3)	
Other	10 (4.4)	4 (3.8)	6 (4.9)	
Before CRRT initiation				
Invasive Mechanical ventilation, *n* (%)	149 (65.6)	67 (64.4)	82 (66.7)	0.78
Vasopressor support, *n* (%)	138 (60.8)	54 (51.9)	84 (68.3)	0.014
APACHE II score	23.6 ± 7.0	22.7 ± 6.6	24.7 ± 7.2	0.034
SOFA score	10.5 ± 3.8	9.5 ± 4.0	11.4 ± 3.4	<0.001
Laboratory before CRRT				
White blood cells (× 10^9^/L)	12.6 ±7.6	12.2 ± 6.4	12.9 ± 8.5	0.50
Platelets (× 10^9^/L)	94 [48, 171]	113.5 [55.5, 184]	77.0 [41.0, 151.0]	0.014
Hemoglobin (g/L)	93.7 ± 26.3	96.7 ± 29.3	91.1 ± 23.3	0.11
Albumin (g/L)	25.7 ± 4.7	26.1 ± 4.8	25.2 ±4.8	0.19
^**^eGFR mL/min/1.73 m^2^	20.4 [12.7, 35.2]	17.4 [12.0, 26.7]	25.2 [14.6, 42.1]	0.002
Creatinine (μmmol/L)	246.6 [155.6, 394.0]	296.4 [216.6, 435.8]	222.0 [133.1, 334.1]	0.001
Lactate (mmol/L)	2.2 [1.4, 5.2]	1.7 [1.3, 3.6]	2.7 [1.7, 6.2]	<0.001
CFB before CRRT (ml)	3,980 [1,910, 8,379]	4,016 [1,928, 7,808]	3,940 [1,410, 8,399]	0.73
Weight-adjusted CFB (%) before CRRT	5.9 [2.7, 12.8]	5.6 [2.8, 10.5]	6.7 [2.4, 14.3]	0.36
Duration of treatment				
Cumulative fluid balance, ml				
48h	204 [−1,400–2,203]	204 [−1,505–1,491]	379 [−1,394–3,274]	0.36
72h	367 [−2,029–2,559]	339 [−2,055–2,049]	403 [−2,029–3,811]	0.12
Weight-adjusted CFB				
48h	0.2 [−2.0–2.9]	0.2 [−2.0–2.5]	0.5 [−2.0–5.1]	0.36
72h	0.6 [−2.8–4.3]	0.4 [−3.0–3.4]	0.8 [−2.7–6.1]	0.42

* *Based on SCr before hospitalization*.

** *Based on SCr at CRRT initiation*.

### Primary and Secondary Outcomes

**Figure 2:**
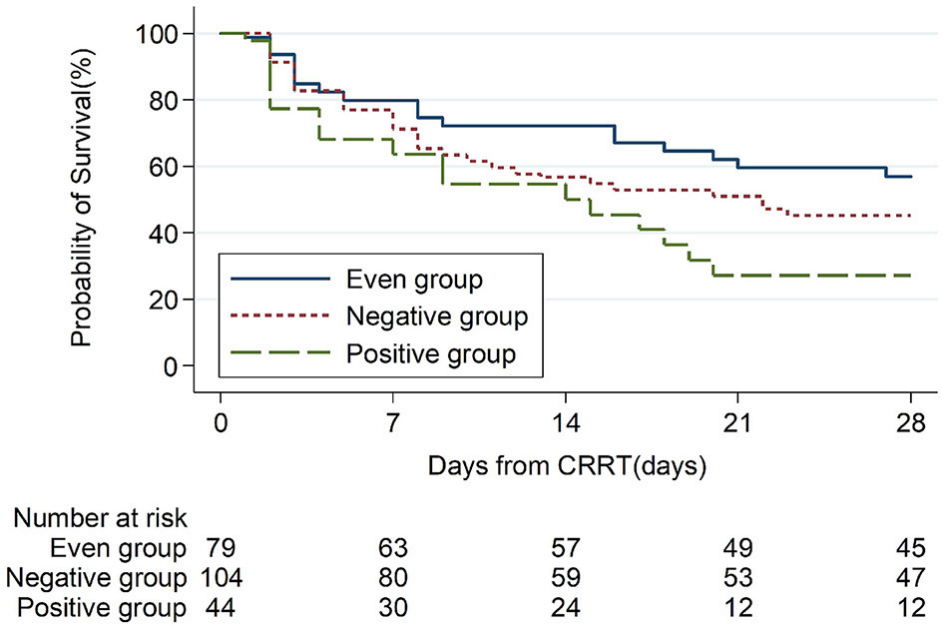
Kaplan Meier analysis of overall survival from baseline (CRRT initiation, day-0) to day-28 in patients with different CFB status.

The 28-day mortality was significantly lower in the even FB group (43.0%, *p* = 0.007, Table 3) than in the positive FB group (72.7%) and the negative FB group (54.8%). A Kaplan–Meier analysis and the log-rank test (Figure 2) indicated significantly longer survival in the even FB group than in the positive FB group, but no significant difference between the even FB and negative FB groups.

**Table 3:** Primary and secondary outcome.

**Outcome**	**All *n =* 227**	**Negative FB (*n =* 104)**	**Even FB (*n =* 79)**	**Positive FB (*n =* 44)**	***p*** **value**
Primary					
Death at 28 days, *n* (%)	123 (54.2)	57 (54.8)	34 (43.0)	32 (72.7)	0.007
Secondary					
Death at 60 days	144 (63.4)	66 (63.5)	45 (57.0)	33 (75.0)	0.13
RRT among survivors, *n*/total (%)	37/104 (35.6)	16/49 (34.0)	16/45 (35.6)	5/12 (41.7)	0.89
MV-free days in survivors	19 [11, 24]	20 [11, 26]	18 [12, 24]	13 [8, 21]	0.16
Vasopressor-free days	24 [14, 27]	25 [23, 28]	20 [0, 26]	24 [19, 27]	0.89
Length of ICU stay					
Survivors	22 [13.5, 44]	21 [15, 34]	26 [13, 48]	20 [11, 35]	0.31
Non-survivors	8 [3, 15]	8 [4, 15]	8 [3, 16.5]	8 [2, 14.75]	0.70

*MV, mechanical ventilation; ICU, Intensive Care Unit; RRT, Renal Replacement Therapy*.

**Figure 3:**
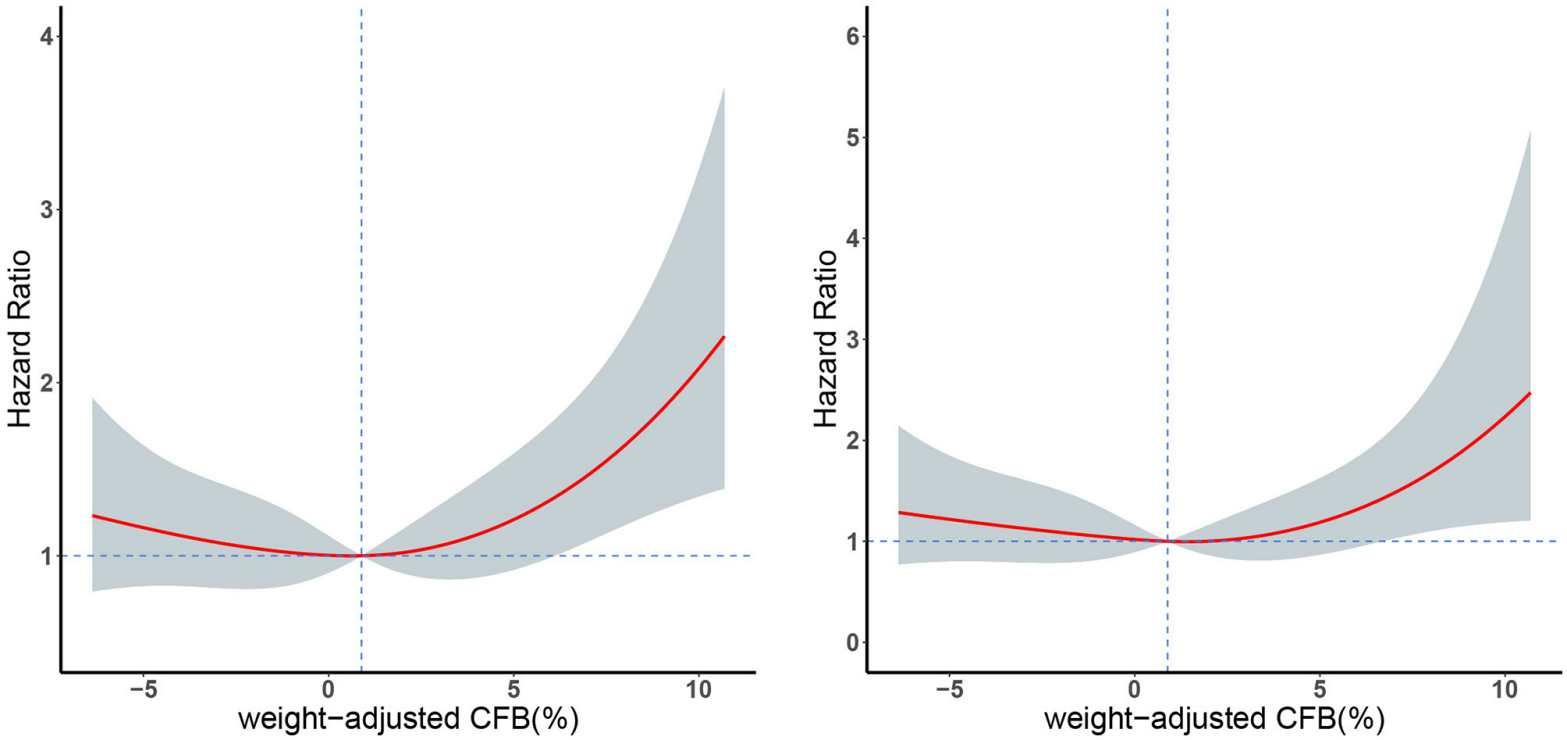
Restricted cubic spline plots of 28-day mortality in patients with different weight-adjusted CFB in an unadjusted Cox model analysis (left) and an adjusted Cox model analysis (right). Red line: hazard ratio, shaded area: 95% CI.

We initially used the univariate Cox models to identify the factors associated with all-cause mortality. Relative to the even FB group, the positive FB group (but not the negative FB group) had an increased adjusted *HR* for mortality in all the Cox regression models (Table 4). In addition, the multivariate Cox model indicated that the 28-day mortality was significantly associated with the female gender, higher SOFA score, and higher eGFR (Table 4). We then used a restricted cubic spline procedure to examine the relationship of the *HR* for 28-day all-cause mortality with CFB, which was treated as a continuous variable (Figure 3). There was a marginal J-shaped association between the CFB and 28-day all-cause mortality in the unadjusted model (*p* for non-linearity = 0.0435), but this relationship was not significant in the adjusted model (*p* for non-linearity = 0.1165).

**Table 4:** Univariate and multivariate Cox model analysis of factors associated with all-cause mortality at day-28 based on CFB status at 48 h.

**Variables**	**Unit**	**Univariate model**		**Multivariate model**					
		**95%CI**	***p*** **value**	**Model 1**		**Model 2**		**Model 3**	
				**95%CI**	***p*** **value**	**95%CI**	***p*** **value**	**95%CI**	***p*** **value**
CFB group	Even FB	1 (reference)		1 (reference)		1 (reference)		1 (reference)	
	Positive FB	2.26 (1.39–3.67)	0.001	2.44 (1.49–3.97)	<0.001	2.09 (1.27–3.43)	0.004	2.30 (1.27–4.17)	0.006
	Negative FB	1.43 (0.93–2.18)	0.10	1.22 (0.78–1.91)	0.38	1.23 (0.77–1.95)	0.39	1.46 (0.88–2.44)	0.15
Age	per 1 year older	1.00 (0.99–1.01)	0.54	1.01 (0.99–1.02)	0.35	1.01 (0.99–1.02)	0.17	1.01 (1.00–1.02)	0.29
Male	vs. Female	0.58 (0.41–0.83)	0.003	0.60 (0.41–0.87)	0.007	0.59 (0.40–0.87)	0.008	0.50 (0.33–0.77)	0.002
BMI	per 1 kg/m^2^	0.95 (0.92–0.99)	0.026	0.95 (0.91–0.99)	0.026	0.95 (0.91–0.99)	0.034	0.97 (0.92–1.02)	0.17
APACHE II score	per 1 pt. increase	1.03 (1.00–1.05)	0.031			1.01 (0.98–1.04)	0.51	1.01 (0.98–1.04)	0.62
SOFA score	per 1 pt. increase	1.10 (1.05–1.16)	<0.001			1.10 (1.04–1.16)	0.002	1.10 (1.03–1.18)	0.003
Septic shock	vs. No	2.71 (1.60–4.59)	<0.001					0.89 (0.57–1.38)	0.59
Chronic kidney disease	vs. No	0.58 (0.37–0.92)	0.022					1.26 (0.73–2.17)	0.41
Hospital–acquired infection	vs. Community–acquired infection	1.50 (1.05–2.14)	0.025					1.53 (0.97–2.43)	0.07
eGFR	per 1ml/min/1.73 m^2^ increase	1.01 (1.00–1.02)	0.009					1.01 (1.00–1.02)	0.017
Lactate	per 1 mmol/l increase	1.06 (1.02–1.10)	0.001					1.02 (0.98–1.07)	0.34
Weight–adjusted CFB (%) before CRRT	per 1% increase	1.02 (1.00–1.04)	0.024					0.99 (0.97–1.01)	0.42

In addition, the three groups had no significant differences in all the four secondary outcome measures—RRT dependence in survivors, vasopressor-free days, mechanical ventilation-free days, and length of ICU stay (Table 3).

Additionally, we collected detailed data on the cause of death (Table 5). Septic shock was the most common cause of death, followed by refractory cardiogenic shock.

**Table 5:** The cause of death.

**Death of cause**	**All *n =* 123**	**Negative FB (*n =* 57)**	**Even FB (*n =* 34)**	**Positive FB (*n =* 32)**	***p*** **value**
Cardiovascular—no. (%)					
Septic shock	41 (33.3)	18 (31.6)	8 (23.5)	15 (46.9)	0.12
Refractory cardiogenic shock	20 (16.3)	12 (21.1)	5 (14.7)	3 (9.4)	0.33
Hypovolemia (bleeding)	15 (12.2)	8 (14.0)	4 (11.8)	3 (9.4)	0.81
Respiratory—no. (%)					
Refractory hypoxia due to ARDS	14 (11.4)	9 (15.8)	2 (5.9)	3 (9.4)	0.33
Pulmonary hemorrhage	5 (4.1)	2 (5.9)	2 (3.5)	1 (3.1)	0.82
Neurological—no. (%)					
Intracranial hemorrhage	2 (1.6)	0 (0.0)	2 (5.9)	0 (0.0)	0.07
Hypoxic encephalopathy	1 (0.8)	0 (0.0)	1 (2.9)	0 (0.0)	0.27
Brain death	4 (3.2)	0 (0.0)	2 (5.9)	2 (6.3)	0.17
Metabolic—no. (%)					
Liver failure	8 (6.5)	2 (3.5)	5 (14.7)	1 (3.1)	0.07
Abandonment of treatment—no. (%)	13 (10.6)	6 (10.5)	3 (8.8)	4 (12.5)	0.89

### Sensitivity Analysis

A logistic regression, with even FB as the comparator, indicated that positive FB was associated with the 28-day mortality (a*OR*: 3.68, 95% *CI*, 1.34–10.13; Table 6). This finding confirmed the robustness of our results.

**Table 6:** Univariate and multivariate logistic model analysis of factors associated with all-cause mortality at day-28 based on CFB status at 48h.

**Variable**	**Unit**	**Univariate model**		**Multivariate model**	
		**OR (95%CI)**	***P*** **-value**	**aOR (95%CI)**	***P-*** **value**
CFB group	Even FB	1 (reference)			
	Positive FB	3.53 (1.59–7.85)	0.002	3.68 (1.34–10.13)	0.012
	Negative FB	1.61 (0.89–2.89)	0.12	1.80 (0.84–3.89)	0.16
Age	per 1 year older	1.01 (0.99–1.03)	0.14	1.02 (0.99–1.04)	0.06
Male	vs. Female	0.41 (0.23–0.73)	0.002	0.25 (0.20–0.53)	<0.001
BMI	per 1 kg/m^2^	0.93 (0.87–0.99)	0.024	0.93 (0.86–1.02)	0.11
APACHE II score	per 1 pt. increase	1.04 (1.00–1.08)	0.036	1.03 (0.97–1.09)	0.29
SOFA score	per 1 pt. increase	1.15 (1.07–1.24)	<0.001	1.15 (1.04–1.27)	0.008
Septic shock	vs. No	4.01 (2.08–7.75)	<0.001	2.64 (1.20–5.80)	0.016
Chronic kidney disease	vs. No	0.47 (0.25–0.87)	0.016	1.11 (0.51–2.42)	0.80
Hospital-acquired infection	vs. Community–acquired infection	2.05 (1.20–3.51)	0.009	2.40 (1.18–4.89)	0.016
eGFR	per 1 ml/min/1.73 m^2^ increase	1.01 (1.00–1.03)	0.057	1.01 (0.99–1.03)	0.18
Lactate	per 1 mmol/l increase	1.13 (1.04–1.21)	0.002	1.09 (0.99–1.20)	0.09
Weight-adjusted CFB (%) before CRRT	per 1% increase	1.01 (0.99–1.04)	0.29	0.97 (0.94–1.00)	0.051

To account for the potential survivorship bias, we used the univariate and multivariate Cox models to analyze the 207 patients who survived beyond 72 h to assess the effect of FB on the 28-day mortality (Table 7). Similar to the above results, the positive FB group had greater 28-day mortality than the even FB group, but there was no significant difference between the even FB and negative FB groups.

**Table 7:** Univariate and multivariate Cox model analysis of factors associated with all-cause mortality at day-28 based on CFB status at 72 h.

**Variable**	**Unit**	**Univariate model**		**Multivariate model**	
		**HR (95%CI)**	***P*** **–value**	**aHR (95%CI)**	***P*** **–value**
CFB group	Even FB	1 (reference)			
	Positive FB	2.81 (1.67–4.73)	<0.001	2.11 (1.17–3.83)	0.014
	Negative FB	1.37 (0.87–2.17)	0.18	0.99 (0.61–1.62)	0.97
Age	per 1 year older	1.09 (0.99–1.01)	0.60	1.01 (1.00–1.03)	0.03
Male	vs. Female	0.51 (0.35–0.75)	0.001	0.44 (0.29–0.68)	<0.001
BMI	per 1 kg/m^2^	0.95 (0.91–0.99)	0.021	0.96 (0.90–1.01)	0.13
APACHE II score	per 1 pt. increase	1.02 (0.99–1.05)	0.13	0.99 (0.96–1.03)	0.58
SOFA score	per 1 pt. increase	1.10 (1.05–1.16)	<0.001	1.13 (1.05–1.21)	0.001
Septic shock	vs. No	2.84 (1.60–5.07)	<0.001	1.52 (0.78–2.94)	0.22
eGFR	per 1 ml/min/1.73 m^2^ increase	1.01 (1.00–1.02)	0.013	1.01 (1.00–1.02)	0.024
Lactate	per 1 mmol/l increase	1.06 (1.02–1.10)	0.007	1.03 (0.98–1.08)	0.22
Weight-adjusted CFB (%) before CRRT	per 1% increase	1.03 (1.01–1.04)	0.006	1.01 (0.98–1.03)	0.82

Furthermore, the positive FB group still had a higher risk of death at 28 days when we used the propensity score as a covariate in the two other sensitivity analyses (Tables 8, 9).

**Table 8:** Univariate and multivariate Cox model analysis of factors associated with all-cause mortality at day-28–propensity score Model 1.

**Variable**	**Unit**	**Univariate model**		**Multivariate model**	
		**HR (95%CI)**	***P*** **–value**	**aHR (95%CI)**	***P*** **–value**
CFB group	Even FB	1 (reference)			
	Positive FB	2.26 (1.39–3.67)	0.001	1.77 (1.04–3.00)	0.034
	Negative FB	1.43 (0.93–2.18)	0.10	1.33 (0.86–2.05)	0.20
Propensity score	per 1 pt	0.12 (0.03–0.50)	0.003	0.20 (0.04–0,87)	0.033

**Table 9:** Univariate and multivariate Cox model analysis of factors associated with all-cause mortality at day-28–propensity score Model 2.

**Variable**	**Unit**	**Univariate model**		**Multivariate model**	
		**HR (95%CI)**	***P*** **-value**	**aHR (95%CI)**	***P*** **-value**
CFB group	Even FB	1 (reference)			
	Positive FB	2.26 (1.39–3.67)	0.001	1.89 (1.07–3.33)	0.027
	Negative FB	1.43 (0.93–2.18)	0.10	1.37 (0.89–2.11)	0.16
Propensity score	per 1 pt	0.31 (0.10–0.94)	0.039	0.57 (0.16–2.01)	0.38

### Subgroup Analysis

We performed a subgroup analysis using the univariate and multivariate Cox models to assess the association of CFB with 28-day mortality in the patients who had septic shock (Table 10). The univariate analysis showed that the positive FB and negative FB groups had higher mortality rates than the even FB group, but only the positive FB group had a greater mortality rate in the multivariate analysis. We conducted another subgroup analysis in the patients with fluid overload at CRRT initiation, in which the fluid overload was defined as a weight-adjusted CFB (from hospital admission to CRRT initiation) more than 5%. In line with the previous results, the positive FB group had a significantly higher mortality rate than the even FB group (Table 11).

**Table 10:** Univariate and multivariate Cox model analysis of factors associated with all-cause mortality at day-28 in patients with septic shock based on CFB status at 48 h.

**Variable**	**Unit**	**Univariate model**		**Multivariate model**	
		**HR (95%CI)**	***P*** **-value**	**aHR (95%CI)**	***P*** **–value**
CFB group	Even FB	1 (reference)			
	Positive FB	2.01 (1.26–3.49)	0.004	2.30 (1.24–4.29)	0.009
	Negative FB	1.74 (1.09–2.78)	0.021	1.46 (0.83–2.56)	0.19
Age	per 1 year older	1.01 (0.99–1.02)	0.39	1.01 (0.99–1.02)	0.20
Male	vs. Female	0.55 (0.38–0.81)	0.002	0.52 (0.33–0.82)	0.005
BMI	per 1 kg/m^2^	0.97 (0.93–1.01)	0.16	0.98 (0.93–1.04)	0.53
APACHE II score	per 1 pt. increase	1.01 (0.98–1.04)	0.47	1.00 (0.96–1.03)	0.78
SOFA score	per 1 pt. increase	1.07 (1.01–1.12)	0.017	1.08 (1.01–1.16)	0.022
Hospital-acquired infection	vs. Community–acquired infection	1.28 (0.88–1.87)	0.20	1.40 (0.84–2.34)	0.20
eGFR	per 1ml/min/1.73m^2^ increase	1.01 (1.00–1.02)	0.16	1.01 (0.99–1.02)	0.07
Lactate	per 1mmol/l increase	1.05 (1.01–1.10)	0.018	1.02 (0.97–1.07)	0.46
Weight-adjusted CFB (%) before CRRT	per 1% increase	1.02 (1.00–1.13)	0.09	1.00 (0.97–1.02)	0.78

**Table 11:** Univariate and multivariate Cox model analysis of factors associated with all-cause mortality at day-28 in patients with fluid overload based on CFB status at 48 h.

**Variable**	**Unit**	**Univariate model**		**Multivariate model**	
		**HR (95%CI)**	***P*** **–value**	**aHR (95%CI)**	***P*** **–value**
CFB group	Even FB	1 (reference)			
	Positive FB	2.67 (1.41–5.08)	0.003	2.33 (1.19–4.55)	0.013
	Negative FB	1.81 (0.99–3.31)	0.054	1.15 (0.58–2.27)	0.69
Age	per 1 year older	1.00 (0.99–1.02)	0.90	1.01 (0.99–1.02)	0.48
Male	vs. Female	0.50 (0.31–0.81)	0.005	0.45 (0.26–0.77)	0.004
APACHE II score	per 1 pt. increase	1.03 (0.99–1.06)	0.07	1.01 (0.98–1.05)	0.50
SOFA score	per 1 pt. increase	1.08 (1.02–1.15)	0.009	1.08 (1.00–1.17)	0.042
Septic shock	vs. No	2.71 (1.09–6.73)	0.032	1.41 (0.54–3.70)	0.49
Lactate	per 1mmol/l increase	1.09 (1.04–1.15)	0.001	1.08 (1.02–1.14)	0.007

## Discussion

### Key Findings

We assessed the prognostic value of early CFB after CRRT initiation in a homogeneous population of patients with septic AKI. The results indicated that the patients with positive FB had a higher risk of 28-day mortality than the patients with even FB, but the patients with negative FB and even FB had similar risks of 28-day mortality. Furthermore, the survivors in the positive FB, even FB, and negative FB groups had no significant differences in the RRT dependence, mechanical ventilation-free days, vasopressor-free days, or length of ICU stay.

### Comparisons With the Previous Studies

One of the main concerns in the treatment of patients with AKI undergoing CRRT is providing precise control of FB (27, 28). Hyung et al. reported that the CFB at 24 and 72 h after the initiation of CRRT were significantly higher in the 28-day non-survivors than survivors (19); however, we found no significant difference in CFB between the survivor and non-survivor groups. One possible reason for our disparate results is that we included the patients who had simultaneous sepsis and AKI, and fluid resuscitation was a major step in the management of these patients. The previous studies of patients with AKI reported that a positive FB after CRRT initiation increased the risk of adverse outcomes (18, 20). A secondary analysis of the Randomized Evaluation of Normal versus Augmented Level (RENAL) trial clearly showed that the presence of a mean daily positive FB after RRT initiation, even within the first 48 h of RRT, was independently associated with the higher mortality in the critically ill patients with severe AKI (18). A prospective cohort study that assessed the association of FB in the 7 days after RRT initiation reported similar results (20). The findings of our study are consistent with these previous studies, in that a positive FB is associated with an increased risk of death in the patients with septic AKI undergoing CRRT. A difference in our study is that we used an even FB group (rather than the negative FB group) as a comparator. Additionally, the prior studies measured volume status as mean daily FB after CRRT initiation, whereas we used CFB during the first 48 and 72 h after onset of CRRT. Despite this methodologic difference, we found similar associations between the positive FB and unfavorable outcomes. Thus, the determination of appropriate fluid management during the RRT is an important topic for future clinical trials.

A recent large retrospective study by Balakumar et al. (26) demonstrated that positive FB and negative FB before RRT initiation were both associated with higher mortality relative to even FB. Furthermore, the present study found that the 28-day mortality in the negative FB group was higher than in the even FB group; however, our univariate and multivariate Cox analysis indicated that negative FB did not significantly increase the risk of all-cause mortality at day-28 relative to even FB. One possible interpretation of these results is that fluid removal using the RRT may provide benefits to some patients because we calculated CFB during the first 48 h after the CRRT initiation. In contrast, Balakumar et al. (26) calculated CFB before RRT initiation. Moreover, we found a marginal J-shaped relationship (rather than a linear relationship) between the 48 h CFB and 28-day mortality in an unadjusted model, although this relationship was not significant in the adjusted model. It is likely that our finding of a negative effect of FB during RRT differed from some previous studies (29, 30) because of differences in the characteristics of patients. In our study, 60% of the patients received vasopressor support at CRRT initiation and 80% experienced septic shock during their ICU stays. The safe achievement of a negative FB during the late phases of septic shock is considered an effective strategy of fluid management (31), and active fluid removal using RRT may cause hemodynamic instability and lead to a worse outcome. Our results may suggest that achieving a negative FB rapidly after CRRT initiation is potentially harmful in patients with septic AKI. Thus, further research is needed to elucidate the benefits and harms associated with the negative FB in these patients.

Our study also evaluated the relationships of CFB and renal recovery in critically ill adults with septic AKI. We found that negative FB and positive FB after CRRT initiation were unrelated to the renal recovery, consistent with the prior studies (20, 26). Our study, thus, confirmed the recent findings that a substantial percentage of RRT-requiring AKI survivors remain dependent on the RRT even after the acute phase of their illness has resolved (20, 32, 33). This serves as a reminder of the need for the measures that protect and restore kidney functions during and after an episode of AKI that necessitates RRT.

### Strengths and Limitations

To our knowledge, this is the first study to use an even FB group as a comparator to evaluate the association of CFB status during the CRRT and mortality in patients with septic AKI. Nonetheless, there were several limitations in the current study. First, our study was retrospective, conducted in a single center, and the sample size was relatively small. Thus, the selection bias was possible and we were unable to make causal inferences regarding the FB and outcomes. However, we used propensity scores in the sensitivity analysis to reduce the effects of outcome-selection bias. Second, our choice of measuring CFB at 48 h after initiation of CRRT was somewhat arbitrary. Nevertheless, the timing of this assessment varies among the studies, and there is no consensus on the optimal time for this measurement. In addition, our sensitivity analysis of the patients who survived at least 3 days after CRRT initiation produced similar results. Third, although we adjusted for confounding using robust multivariable regression analysis, residual confounding by unknown factors is possible.

## Conclusion

Our study of critically ill patients with septic AKI indicated that the patients with positive FB after CRRT initiation had an increased risk of 28-day mortality relative to the patients with even FB. Although not statistically significant, we noted a trend toward higher mortality in the patients with negative FB compared with those with even FB, a topic that might warrant further investigation.

## Data Availability Statement

The original contributions presented in the study are included in the article/supplementary material, further inquiries can be directed to the corresponding author/s.

## Ethics Statement

The studies involving human participants were reviewed and approved by Bioethics Committee of Beijing Friendship Hospital, Capital Medical University. The Ethics Committee waived the requirement of written informed consent for participation.

## Author Contributions

JL and MD designed the study. JL and HZ conducted the statistical analysis, interpreted the results, and critically revised the manuscript. JB, DZ, ZQ, and SL made a substantial contribution to the acquisition of the data. LD conducted the revision for the manuscript. All authors contributed to the manuscript, approved the final version to be considered for publication, and read and approved the final manuscript.

## Funding

This study was funded by the Training Program of the Research Plan of Beijing Hospital Authority (PX2021003).

## Conflict of Interest

The authors declare that the research was conducted in the absence of any commercial or financial relationships that could be construed as a potential conflict of interest.

## Publisher's Note

All claims expressed in this article are solely those of the authors and do not necessarily represent those of their affiliated organizations, or those of the publisher, the editors and the reviewers. Any product that may be evaluated in this article, or claim that may be made by its manufacturer, is not guaranteed or endorsed by the publisher.
